# Long-Term Safety and Efficacy of Renal Denervation: 24-Month Results From the SPYRAL HTN-ON MED Trial

**DOI:** 10.1161/CIRCINTERVENTIONS.125.015194

**Published:** 2025-05-20

**Authors:** David E. Kandzari, Felix Mahfoud, Raymond R. Townsend, Kazuomi Kario, Michael A. Weber, Roland E. Schmieder, Konstantinos Tsioufis, Stuart Pocock, Minglei Liu, Vanessa DeBruin, Sandeep Brar, Michael Böhm

**Affiliations:** 1 Piedmont Heart Institute, Atlanta, GA (D.E.K.).; 2 Department of Cardiology (F.M.), University Heart Center, University Hospital Basel, Switzerland.; 3 Cardiovascular Research Institute Basel (CRIB) (F.M.), University Heart Center, University Hospital Basel, Switzerland.; 4 Perelman School of Medicine, University of Pennsylvania, Philadelphia (R.R.T.).; 5 Department of Cardiovascular Medicine, Jichi Medical University School of Medicine, Tochigi, Japan (K.K.).; 6 SUNY Downstate College of Medicine, New York, NY (M.A.W.).; 7 University Hospital Erlangen, Germany (R.E.S.).; 8 National and Kapodistrian University of Athens Hippocratio Hospital, Athens, Greece (K.T.).; 9 London School of Hygiene and Tropical Medicine, United Kingdom (S.P.).; 10 Medtronic, Santa Rosa, CA (M.L., V.D.B., S.B.).; 11 Universitätsklinikum des Saarlandes, Saarland University, Homburg, Germany (M.B.).

**Keywords:** antihypertensive agents, blood pressure, denervation, hypertension

## Abstract

**BACKGROUND:**

Six-month results from the SPYRAL HTN-ON MED trial (SPYRAL HTN-ON MED Study of Renal Denervation With the Symplicity Spyral Multi-Electrode Renal Denervation System) demonstrated that renal denervation (RDN) reduced office blood pressure (BP), and not 24-hour ambulatory systolic BP, compared with sham control in hypertensive patients. In this prespecified analysis of the ON MED trial, long-term changes in BP, antihypertensive drug use, and safety outcomes through 24 months are compared between RDN and sham control groups.

**METHODS:**

SPYRAL HTN-ON MED is a prospective, randomized, sham-controlled, blinded trial enrolling 337 patients globally from 56 clinical centers. Eligible patients had an office systolic BP of 150 to 180 mm Hg, a diastolic BP ≥90 mm Hg, and a 24-hour ambulatory systolic BP of 140 to 170 mm Hg. Patients were randomized to RDN or a sham control procedure and were prescribed a stable regimen of 1 to 3 antihypertensive medications through 6 months. After 6 months, patients and physicians were unblinded with permitted changes to antihypertensive therapy, and control patients were permitted to cross over. Crossover patients had their last observations carried forward as part of the control group. Statistical analyses were conducted on the population as randomized.

**RESULTS:**

At 24 months, the RDN group experienced significantly greater mean reductions in ambulatory systolic BP (−12.1±15.3 mm Hg [n=176] versus −7.0±13.1 mm Hg [n=33]; difference: −5.7 mm Hg; *P*=0.039) and office systolic BP (−17.4±16.1 mm Hg [n=187] versus −9.0±19.4 mm Hg [n=35]; difference: −8.7 mm Hg; *P*=0.0034) compared with sham controls. At 24 months, antihypertensive medications increased significantly more in the sham group (1.7 versus 2.7) compared with the RDN group (1.8 versus 2.4; *P*=0.046). Sensitivity analyses accounting for missing sham patient BP values due to crossover yielded consistent results in favor of RDN for 24-hour ambulatory (*P*=0.023) and office systolic BP (*P*<0.0001). Clinically adverse events were rare, with no instances of renal artery stenosis through 24 months.

**CONCLUSIONS:**

RDN produced significantly greater ambulatory and office systolic BP reductions at 24 months compared with sham control, despite higher antihypertensive medication use in the control group.

**REGISTRATION:**

URL: https://clinicaltrials.gov; Unique identifier: NCT02439775.

**WHAT IS KNOWN**

- Results from the SPYRAL HTN-ON MED study (SPYRAL HTN-ON MED Study of Renal Denervation With the Symplicity Spyral Multi-Electrode Renal Denervation System) demonstrated that renal denervation reduced office blood pressure compared with sham control at 6 months, and not 24-hour ambulatory blood pressure.

**WHAT THE STUDY ADDS**

- In this prespecified analysis comparing long-term results between renal denervation and sham control patients, the renal denervation group experienced greater reductions in both office and 24-hour ambulatory blood pressures at 24 months compared with the sham control group, despite higher antihypertensive medication use in the sham control group.
- Sensitivity analyses accounting for control patients who crossed over yielded similar results.
- Adverse events remained rare through long-term follow-up.

Hypertension is the most common modifiable risk factor for cardiovascular events.^1,2^ Catheter-based renal denervation (RDN) reduces high blood pressure (BP) by targeting the sympathetic nervous system.^3–6^ Randomized sham-controlled trials have demonstrated the safety and efficacy of RDN in the absence and presence of antihypertensive medications.^7–10^ Results from the SPYRAL HTN-ON MED trial (SPYRAL HTN-ON MED Study of Renal Denervation With the Symplicity Spyral Multi-Electrode Renal Denervation System) showed a significant treatment difference between RDN and sham control groups in office systolic BP at 6 months, but not 24-hour ambulatory systolic BP.^10^ Multiple confounding factors, however, may have influenced results, including documented disproportionate medication increases among sham control patients.^11^

It is important to establish whether RDN yields durable effects in reducing BP through longer-term follow-up. Multiple studies have demonstrated long-term reductions through 3 years and beyond,^12,13^ including a large all-comers, global registry reflecting the real-world population with long-term 3-year follow-up in more than 1200 patients.^14,15^ In the earlier randomized sham-controlled SPYRAL HTN-ON MED Pilot trial, BP reductions among RDN patients were sustained compared with sham control patients over time through 3 years. Moreover, in the randomized, sham-controlled Symplicity HTN-3 trial (SYMPLICITY HTN-3 Renal Denervation in Patients With Uncontrolled Hypertension), reductions in office and 24-hour ambulatory systolic BP were not significantly different at 6 months.^16^ Nonetheless, by 3 years, BP reductions were significantly greater among RDN patients compared with sham control patients.^13^ In this prespecified analysis of the SPYRAL HTN-ON MED trial, we compare long-term changes in BP and antihypertensive medication use and safety results through 24 months between RDN and sham control groups.

## Methods

### Study Design and Patients

The data that support the findings of this study are available upon reasonable request. SPYRAL HTN-ON MED is a global, randomized, blinded, sham-controlled trial assessing the safety and efficacy of catheter-based radiofrequency RDN in patients taking 1 to 3 antihypertensive medications.^17^ Patients between the ages of 20 and 80 years with uncontrolled hypertension, defined as office systolic BP≥150 and <180 mm Hg, office diastolic BP≥90 mm Hg, and 24-hour ambulatory systolic BP≥140 and <170 mm Hg, despite taking 1 to 3 antihypertensive medications, were enrolled. Patients were prescribed a stable regimen (1–3) of standard antihypertensive medications through the primary end point ascertainment at 6 months. The first 106 patients were randomized 1:1 to the RDN or sham control procedure, and the subsequent 231 patients were randomized 2:1 to facilitate recruitment. All patients provided written informed consent, and the trial protocol was approved by all local ethics committees and review boards. The trial was designed in accordance with the Declaration of Helsinki.

### Procedure

The specifics of the RDN and sham procedures have been previously described.^7–10^ The Symplicity SPYRAL multielectrode RDN catheter (Symplicity SPYRAL catheter, Medtronic) and Symplicity G3 RDN RF Generator (Symplicity G3 generator, Medtronic) provide circumferential radiofrequency ablations of the renal arteries and branch vessels between 3 and 8 mm in diameter. Each case was performed by an experienced proceduralist and proctored according to predetermined treatment plans. The sham procedure included a renal angiogram only, and patients were required to remain on the procedure table for a minimum of 20 minutes to help prevent possible unblinding.

### Follow-Up

Patients were followed at 1-, 3-, 6-, 12-, and 24-month post-procedure. Office BP was assessed by blinded trial staff, followed by witnessed pill intake. Assessment of 24-hour ambulatory BP was at 3, 6, 12, and 24 months. Antihypertensive medication adherence was assessed by urine and plasma testing. According to the protocol, antihypertensive medication and dosage changes were prohibited through 6 months unless prespecified criteria were met.^17^ Duplex ultrasound, computerized tomography, or magnetic resonance imaging assessed renal artery anatomy. Sham control patients were permitted to cross over to RDN after the primary end point ascertainment, without having to requalify per trial eligibility criteria. Crossover patient follow-up through 24 months is ongoing.

### Outcomes

The primary efficacy end point was the treatment difference in mean 24-hour ambulatory systolic BP at 6 months post-randomization between RDN and sham control groups using a Bayesian design.^17^ Secondary end points are listed in Table S1. Long-term safety was compared between groups through 24 months including all-cause mortality, myocardial infarction, major bleeding (TIMI), significant embolic event resulting in end-organ damage, renal artery reintervention, vascular complications requiring surgical repair, intervention, thrombin injection, or blood transfusion, hypertensive crisis, stroke, and renal artery stenosis >70%.

### Statistical Analysis

The primary statistical analyses were conducted on the population with evaluable data according to their original randomization. Sham control patients who crossed over to RDN were censored at that time (no data were carried forward). Categorial variables are reported as percentages and counts and were compared between treatment groups using exact binomial tests. Continuous variables are reported as mean±SD. Changes in BP or medication measures were compared between treatment groups using analysis of covariance (ANCOVA), adjusting for baseline measurements. Comparison of the change in estimated glomerular filtration rate (eGFR) was performed using a 2-sample t test. Sensitivity analyses were conducted that were consistent with prior RDN trials reporting long-term follow-up imputing the last observations carried forward for sham control patients who crossed over and underwent RDN,^12,13^ and for sham patients with missing measures due to follow-ups via phone call. In the last observations carried forward analyses, all measures (BP, medications, eGFR, etc) from the last evaluable follow-up were carried forward to the final follow-up. If crossover procedures took place >30 days since the most recent follow-up, measures were reassessed before treatment and carried forward to the final follow-up. Two additional sensitivity analyses were performed. The first used the multiple imputation method utilizing SAS PROC MI (SAS Institute, Research Triangle, NC) to perform a Markov chain Monte Carlo algorithm. One hundred datasets were imputed using treatment groups, age, body mass index, sex, baseline, 3-, 6-, 12-, and 24-month systolic BP and medications (number and burden). The second sensitivity analysis utilized inverse probability censoring weights using treatment groups, age, body mass index, sex, baseline, 3-, 6-, 12-, and 24-month systolic BP, and medications (number and burden). Treatment differences between RDN patients and sham control patients, including imputed values, were ANCOVA-adjusted using baseline BP. Where indicated, treatment differences also accounted for antihypertensive medication burden changes (based on adherence testing) from baseline. Clinical safety events through 24 months were adjudicated by an independent clinical event committee and estimated using the Kaplan-Meier method. For sham patients who crossed over to RDN, safety events were censored at that time. Statistical analysis was performed using SAS for Windows 9.4 (SAS Institute, Research Triangle, NC).

## Results

**Figure 1.**
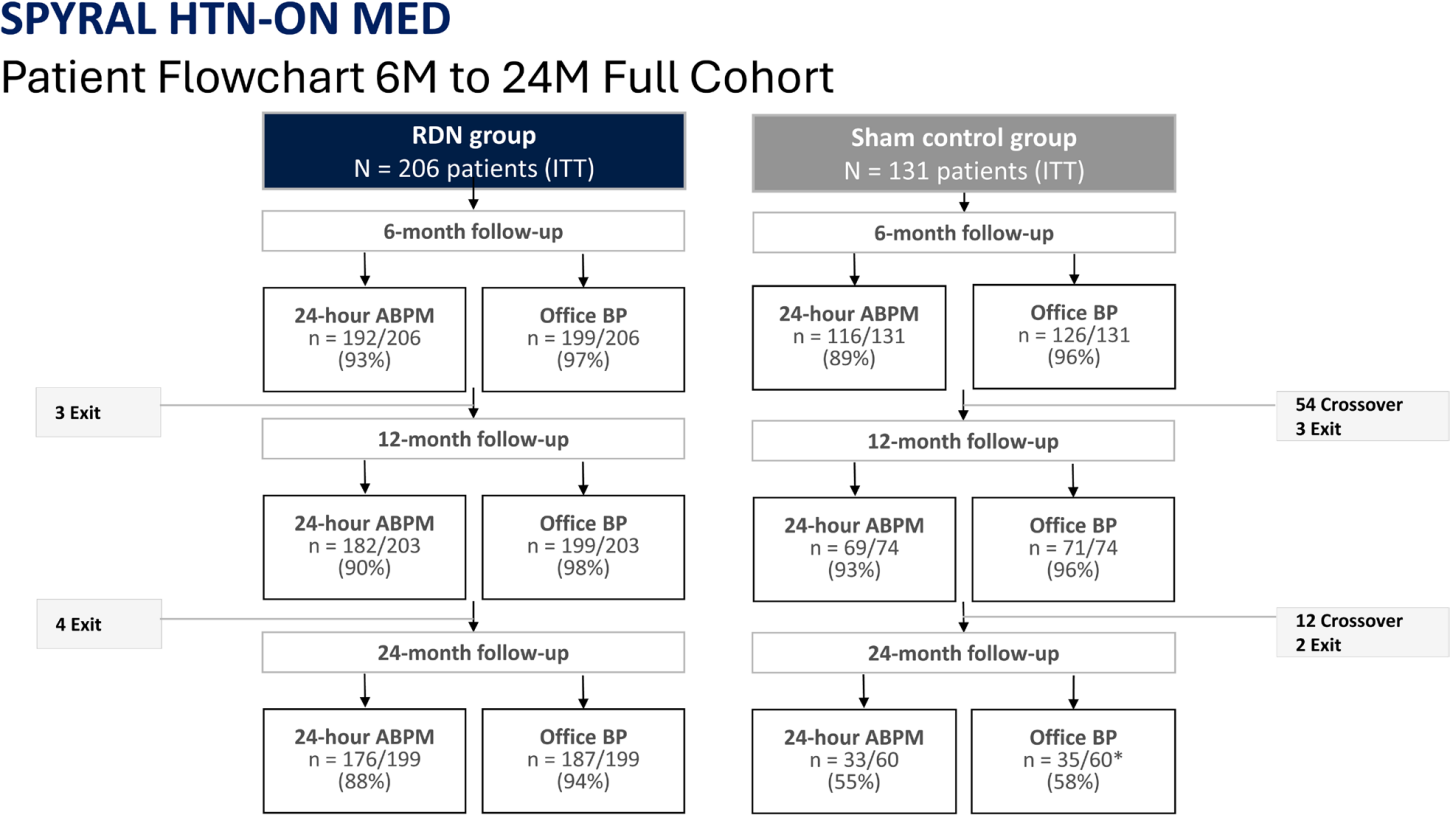
**ON MED patient flowchart through 24 months.** After a 6-month primary end point ascertainment in the SPYRAL HTN-ON MED trial (SPYRAL HTN-ON MED Study of Renal Denervation With the Symplicity Spyral Multi-Electrode Renal Denervation System), sham control patients were permitted to cross over to undergo renal denervation (RDN) irrespective of blood pressure (BP) control. *At 24 months, 23 sham patients were followed via televisits permitted per protocol. Overall follow-up compliance was 97% at 24 months, including televisits. Percentages represent patients with observed outcomes. ABPM indicates 24-hour ambulatory blood pressure measure; and ITT, intention-to-treat.

Between July 22, 2015, and February 15, 2022, 1780 patients were enrolled from 56 clinical centers worldwide. Among enrolled patients, 337 met the eligibility criteria and were randomly assigned to undergo RDN (n=206) or the sham control procedure (n=131; Figure 1). After the primary end point ascertainment at 6 months, patients in the sham control group were allowed to cross over to receive the RDN procedure, with 54 sham patients crossing over after their 6-month follow-up and an additional 12 sham patients crossing over after their 12-month follow-up. The mean time for crossover post-randomization was 241±86 days (8 months; median time to crossover: 201 days).

The baseline characteristics were similar overall between the RDN and sham control patients, including baseline BP measures (Table). The mean 24-hour systolic BP at baseline in the RDN and sham control groups was 150±7 mm Hg and 149±7 mm Hg, respectively. The mean office systolic BP at baseline was 163±8 mm Hg in both groups. Baseline eGFR in the RDN and sham control groups was 81.9±16.8 and 81.9±17.2 mL/min/1.73 m^2^, respectively.^10^

**Table.**
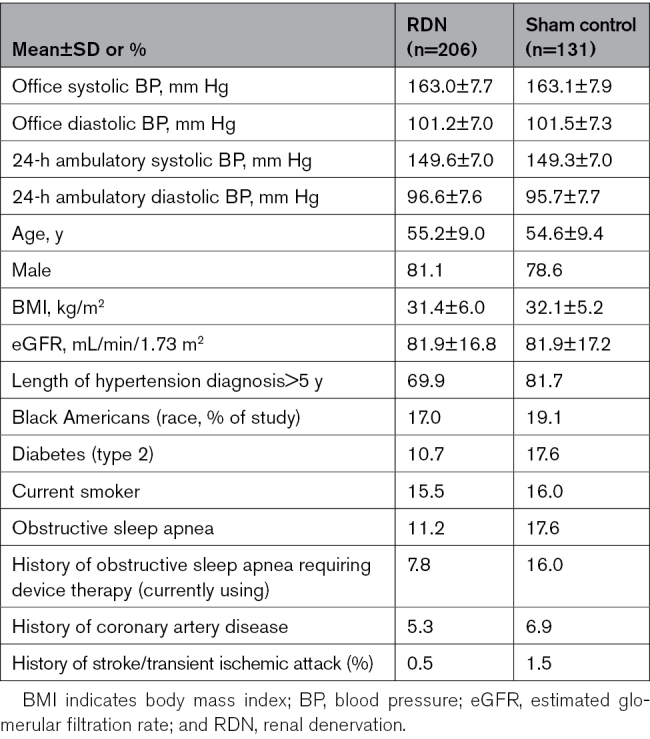
Baseline Patient Characteristics

### Antihypertensive Medication Changes

**Figure 2.**
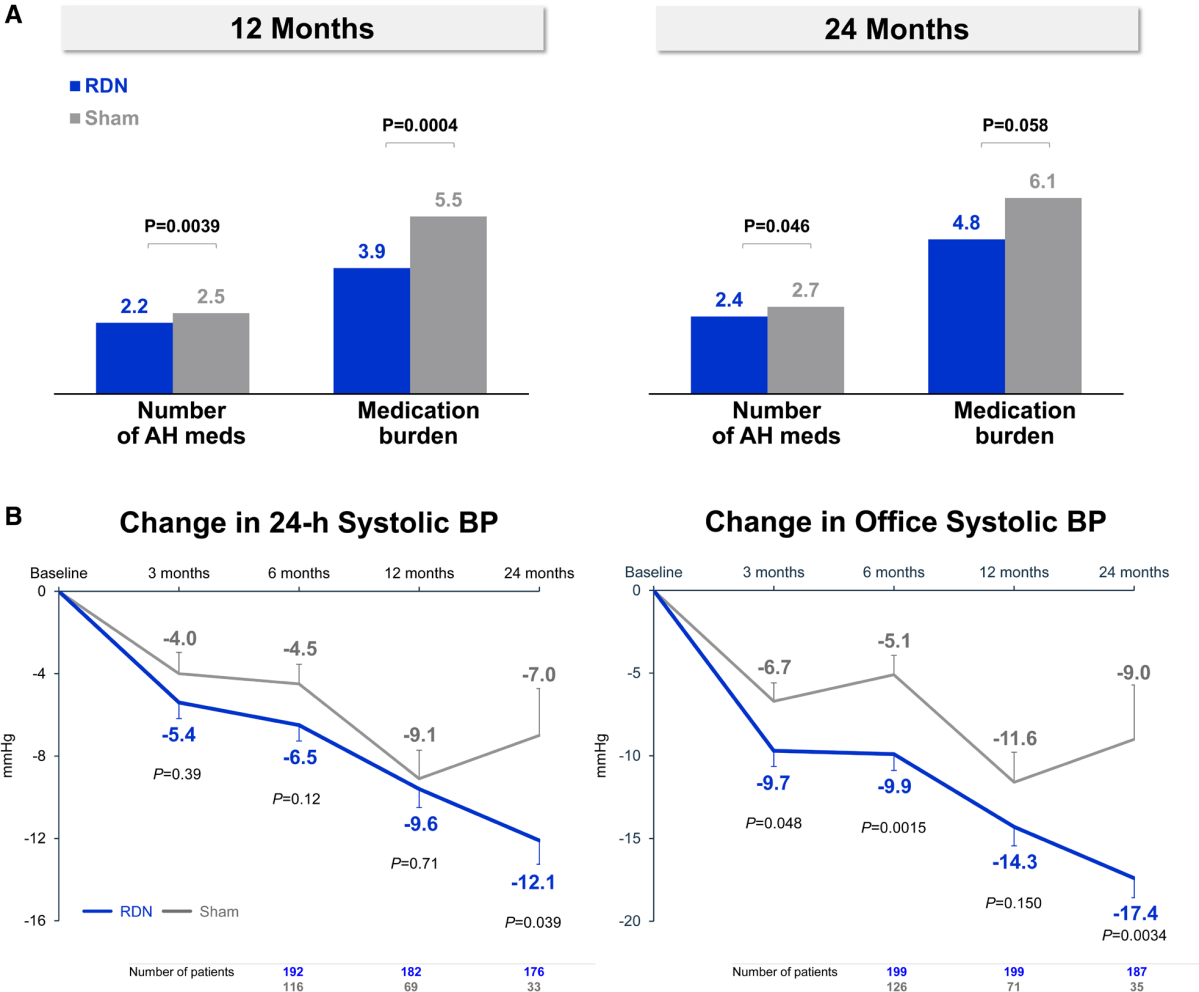
**Antihypertensive (AH) medication and systolic blood pressure (BP) changes through 24 months.** The number of AH medications and medication burden (based on the number, class, and dose)^10^ are plotted (**A**) at 12 and 24 months for renal denervation (RDN; blue) and sham control patients (gray) based on drug testing if available, otherwise prescribed information is used. In (**B**), the changes in the 24-hour ambulatory (**right**) and office systolic BP (**left**) from baseline through 24 months are plotted with the number of patients with available measures indicated below from 6 to 24 months. Follow-up and treatment difference *P* values are analysis of covariance-adjusted for baseline values.

At baseline, the number of medications was 1.8±1.0 versus 1.7±1.0 for the RDN and sham control arms, respectively (*P*=0.29). Based on medication adherence testing, the sham control group took significantly more medications at 12 months (2.2±0.9 versus 2.5±1.1; *P*=0.0039; Figure 2) and 24 months (2.4±1.2 versus 2.7±1.2; *P*=0.046). This resulted in a significantly greater medication burden for the sham control group compared with the RDN group at 12 months (3.9±3.1 versus 5.5±5.2; *P*=0.0004), with a similar trend at 24 months (4.8±4.7 versus 6.1±5.6; *P*=0.058).

### BP Changes

**Figure 3.**
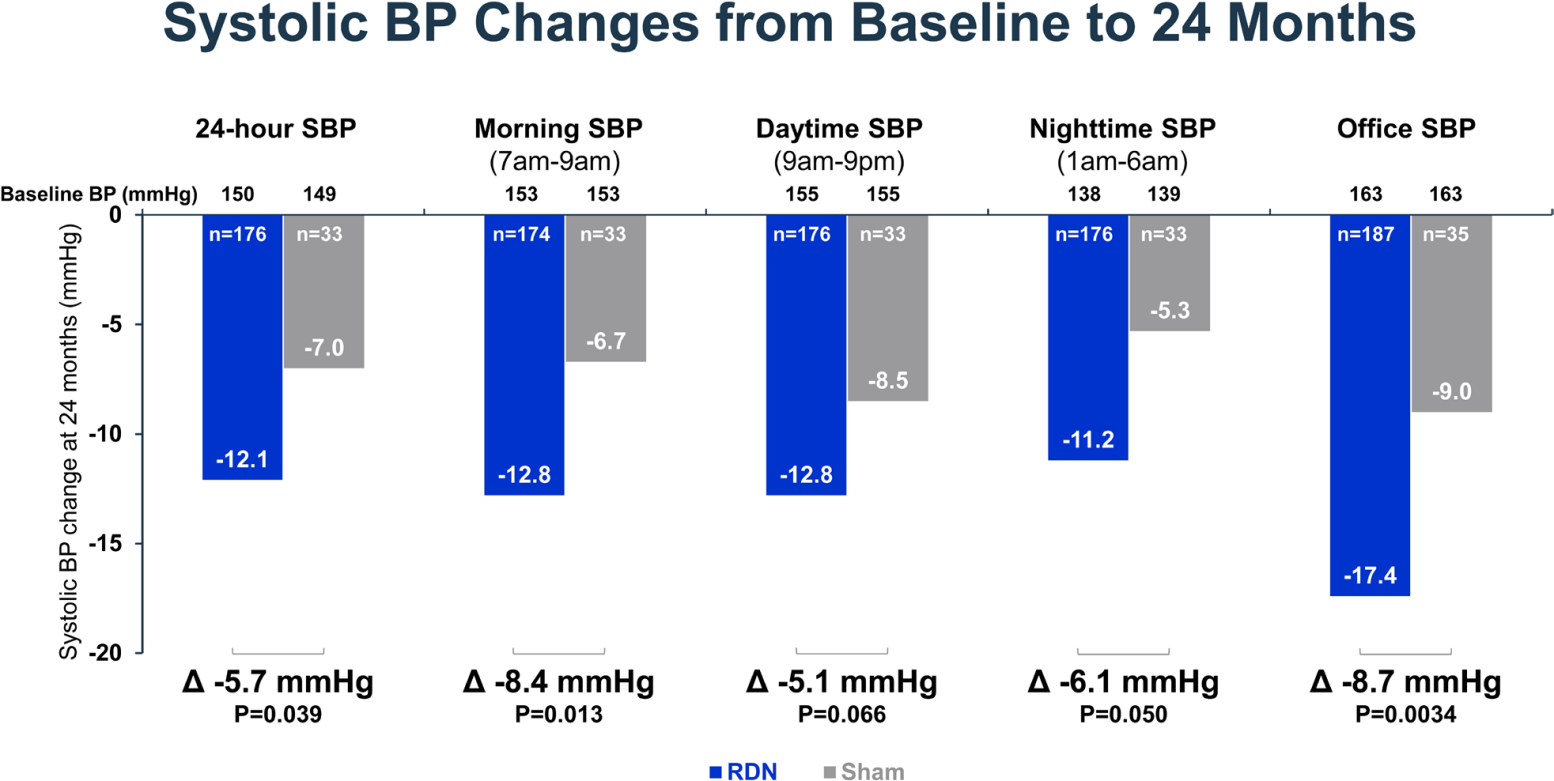
**The change in systolic blood pressure (SBP) measured at 24 months from baseline between renal denervation (RDN) and sham control groups.** The 24-hour ambulatory systolic, morning (7–9 am), daytime (9 am–9 pm), nighttime (1–6 am), and office SBP changes are plotted for RDN (blue) and sham control groups (gray) in patients with available follow-up at 24 months. Comparisons of blood pressure (BP) measures are analysis of covariance-adjusted for baseline BP.

**Figure 4.**
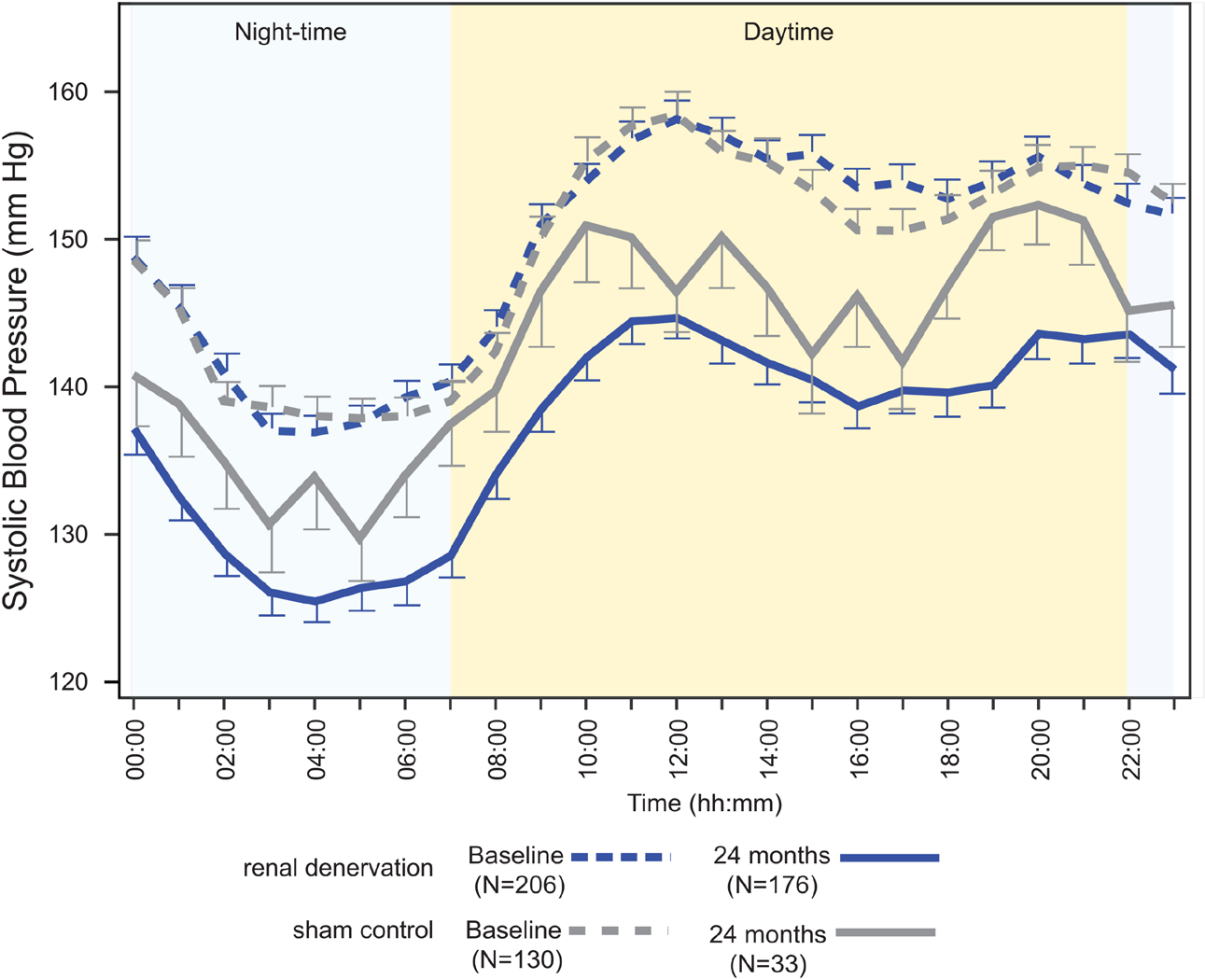
**Hourly ambulatory systolic blood pressure (BP) at baseline and 24 months.** Hourly ambulatory systolic BPs at baseline and 24 months in the renal denervation (blue shades) and sham control groups (gray shades).

Long-term changes in mean 24-hour ambulatory systolic and diastolic BP from baseline demonstrated sustained BP reductions that increased over time in RDN patients (Figure 2 and Figure S1). At 12 months, the ambulatory systolic BP change was similar between groups with −9.6±12.2 mm Hg for RDN patients and −9.1±11.5 mm Hg for sham patients (ANCOVA-adjusted for baseline BP treatment difference: −0.6 mm Hg; *P*=0.71). However, at 24 months, the ambulatory systolic BP change was significantly greater in RDN patients compared with sham control patients (−12.1±15.3 mm Hg versus −7.0±13.1 mm Hg; treatment difference: −5.7 mm Hg; *P*=0.039; Figures 2 and 3). Comparison of hourly changes in 24-hour ambulatory systolic BP between RDN and sham control patients at 24 months reflects greater, sustained reductions throughout the 24-hour period in the RDN group (Figure 4 and Figures S2 and S3). Correspondingly, reductions in ambulatory morning and nighttime systolic BP were significantly greater in the RDN group (Figure 3). Reductions in ambulatory daytime systolic BP were numerically, although not significantly, greater in the RDN group.

**Figure 5.**
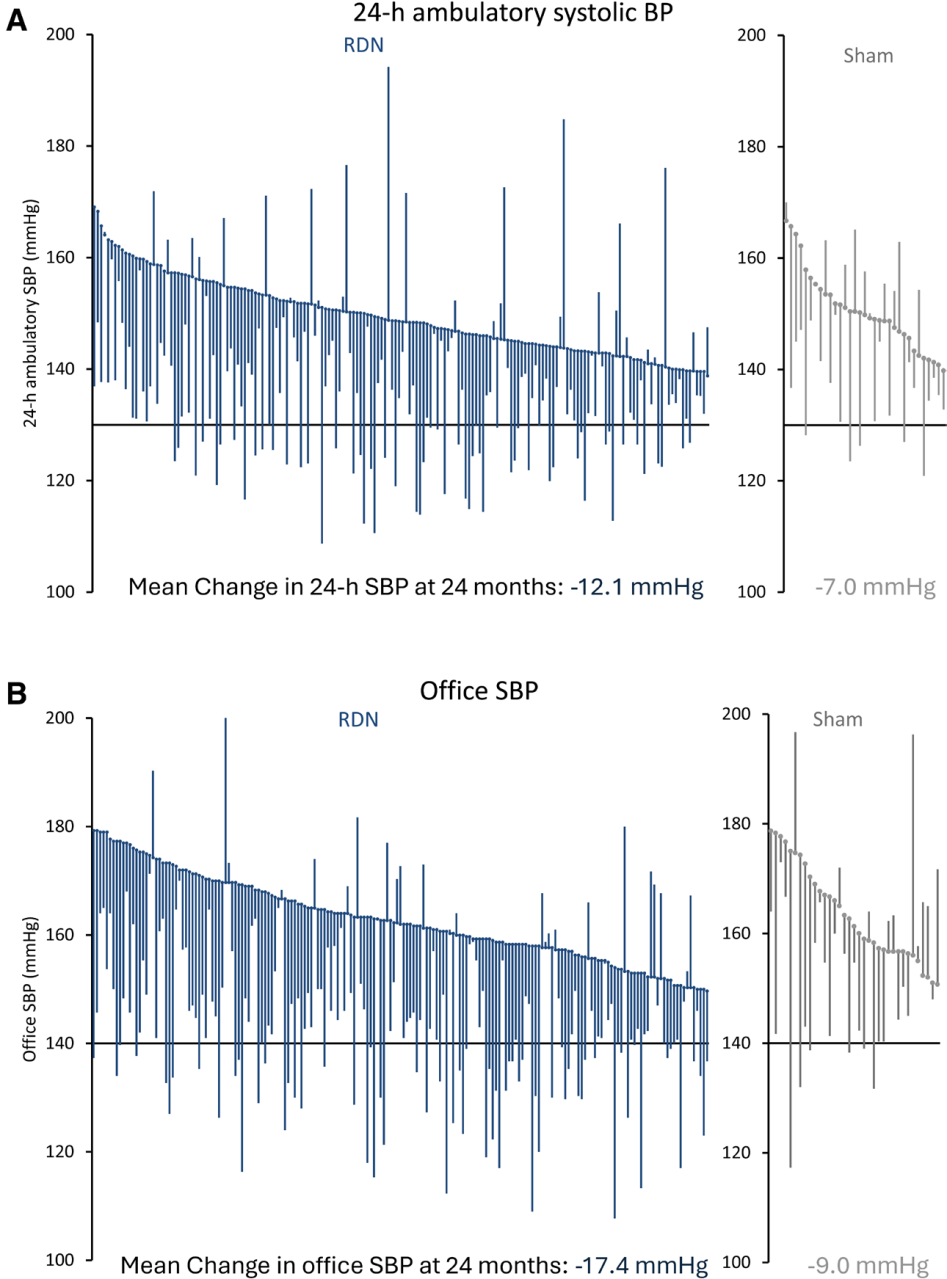
**Patient-level blood pressure (BP) changes from baseline to 24 months.** Individual changes from baseline to 24 months in (**A**) 24-hour ambulatory and (**B**) office systolic BP (SBP) for renal denervation (RDN) patients (blue) and sham control patients (gray) in descending order of baseline SBP. Horizontal line depicts reference BP values.

Similar to 6-month results, long-term changes in office systolic and diastolic BP showed sustained BP reductions that were significantly greater among RDN patients compared with sham control patients at 24 months (Figure 2 and Figure S1). At 12 months, the changes in office systolic BP were −14.3±16.1 mm Hg versus −11.6±15.3 mm Hg among RDN and sham control patients, respectively (treatment difference: −3.1 mm Hg; *P*=0.15). At 24 months, the changes in office systolic BP were −17.4±16.1 mm Hg in the RDN group versus −9.0±19.4 mm Hg in the sham group (treatment difference: −8.7; *P*=0.0034; Figure 3). The change in patient-level 24-hour ambulatory and office systolic BP at 24 months is plotted in Figure 5 for both RDN and sham control groups. Notably, a significantly greater proportion of patients from the RDN group achieved the prespecified secondary end point of a target office systolic BP <140 mm Hg (*P*=0.035). We also assessed the proportion of patients with at least 5, 10, 15, and 20 mm Hg office and 24-hour systolic BP reductions from baseline (Table S2). Two-year systolic BP reductions in various subgroups based on sex, age, body mass index, diabetes status, geography, Black American status, and baseline systolic BP are provided in Figure S4.

**Figure 6.**
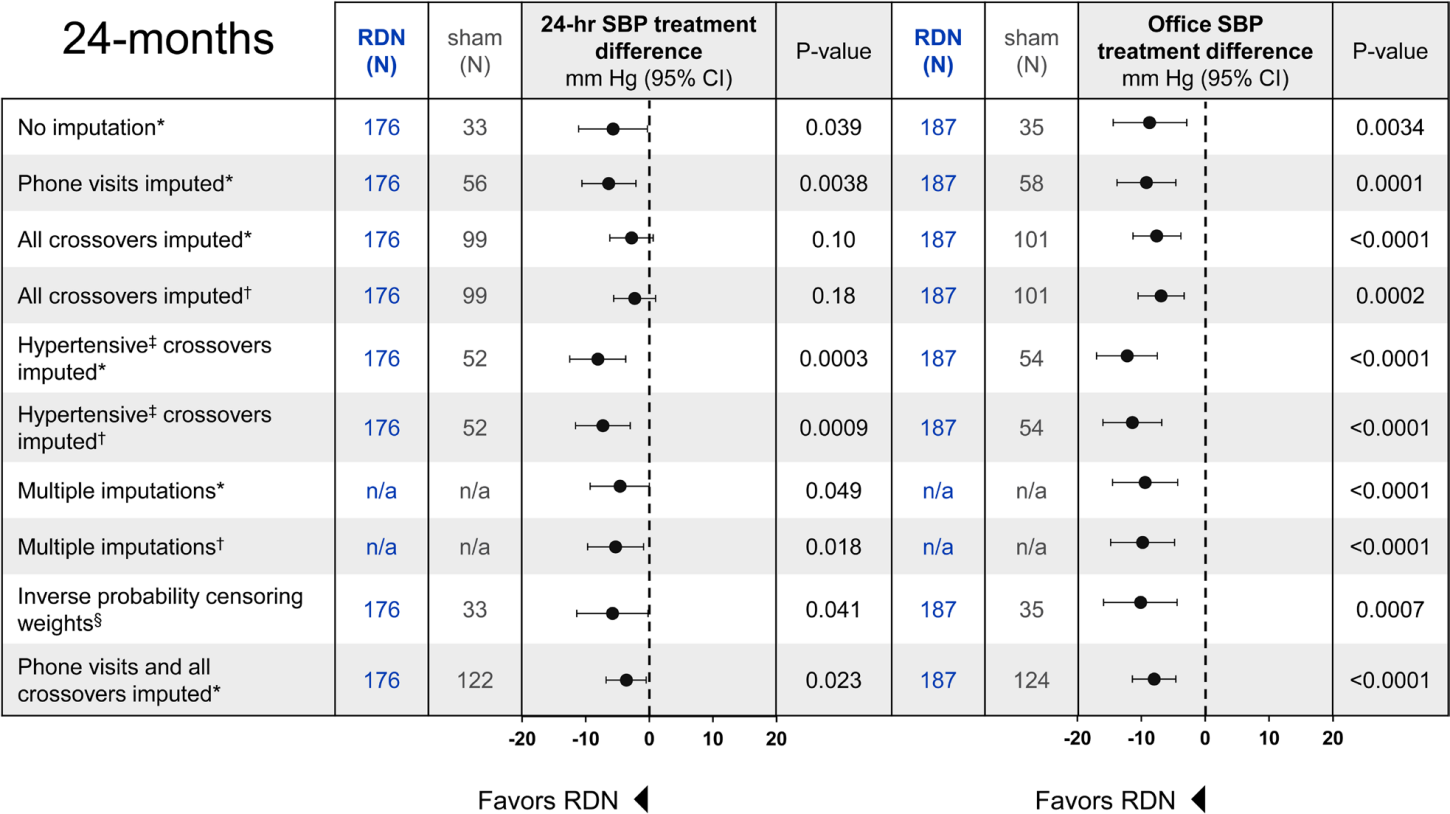
**Summary of sensitivity analyses comparing renal denervation (RDN) and sham control group treatment differences at 24 months.** Forest plot of sensitivity analyses comparing systolic blood pressure (SBP) reductions between RDN and sham control groups at 24 months. *Treatment difference comparisons are analysis of covariance (ANCOVA)-adjusted for baseline BP. †Treatment difference comparisons are ANCOVA-adjusted for baseline BP and the change in medication burden. ‡Hypertensive patients are categorized based on original trial eligibility criteria for SBP (office SBP≥150 mm Hg and 24-hour ambulatory SBP≥140 mm Hg). §Stabilized inverse probability censoring weights were calculated using age, body mass index, sex, baseline BP, medication number, and medication burden.

After the primary end point ascertainment at 6 months, sham control patients were permitted to cross over without requalifying per the original trial BP criteria (see Methods). Additionally, 24-month follow-ups for some sham control patients were permitted to be conducted via phone call. To account for missing BP values among sham control patients due to cross over procedures (50% of sham control patients; n=66) or phone visits (18%; n=23) before 24-month follow-up, several post hoc sensitivity analyses were conducted, including multiple imputations and inverse probability weights (Figure 6 and Figure S5). Results from these sensitivity analyses were broadly consistent, with RDN patients having sustained greater systolic BP reductions compared with sham control patients at 24-month follow-up. A summary of the analyses comparing BP changes in RDN and sham control groups, including an additional multiple imputation analysis for all missing values, is provided in Figure 6.

### Crossover Outcomes

BP changes in crossover patients were separately assessed. Baseline characteristics were similar between sham control patients who crossed over (n=66) and those who did not (n=65), aside from non-crossover patients being more likely to smoke (6.1% versus 26.2%; *P*=0.002). Among 61 sham control patients with available 24-hour ambulatory systolic BP follow-up at 12 months after their crossover RDN procedure, there was a −4.1±14.1 mm Hg change from the procedure (mean pre-crossover measure: 138.2±11.2 mm Hg; *P*=0.027), which equated to a −14.0±13.3 mm Hg change from baseline (*P*<0.0001). There were 64 sham control patients with available office systolic BP follow-up through 12 months after crossover. For office systolic BP, these patients had a −8.5±16.3 mm Hg change from crossover (mean pre-crossover measure: 152.6±13.8 mm Hg; *P*<0.0001), and a −19.1±16.9 mm Hg change from baseline (*P*<0.0001). Comparisons of BP changes between pooled RDN and crossover patients versus non-crossover sham control patients are provided in Table S3. The number of antihypertensive medications and the antihypertensive medication burden between crossover patients and non-crossover patients from baseline to 6-month follow-up, and from 6-month follow-up to final follow-up are provided in Table S4. Notably, non-crossover patients had a significant increase in their antihypertensive medication burden from 6 months to their final follow-up (24 months) compared with crossovers (12 months post-crossover). Moreover, after adjusting for the change in medication burden between patient groups, the reductions in 24-hour ambulatory and office systolic BP in pooled RDN and crossover patients were significantly greater compared with non-crossover patients through long-term follow-up (Table S3).

### Safety Outcomes

Safety events were rare through 24 months, with 1 death in the RDN group and 1 death in the sham control group (Table S5). By 24 months, the change in eGFR was −0.7±11.2 mL/min/1.73 m^2^ in the RDN group and −3.1±7.6 mL/min/1.73 m^2^ in the sham control group (*P*=0.13). There were no instances of renal artery stenosis >70% or reintervention within the renal artery among RDN patients through 24 months.

## Discussion

In this analysis of 24-month results of the SPYRAL HTN-ON MED trial, clinically meaningful and significantly greater reductions in both 24-hour ambulatory and office systolic BP were observed among patients who underwent RDN compared with sham control, despite higher antihypertensive medication use in the sham control group. These findings are consistent with an emerging body of evidence supporting the complementary benefit of RDN with antihypertensive medications and lifestyle interventions to achieve sustained reductions in BP through long-term follow-up.^12,13,18–20^

Thematic of RDN trials, long-term surveillance of BP changes after RDN is challenged by patient unblinding, changes in pharmacotherapy and adherence, BP rise over time, coexisting medical conditions, and sham control patients crossing over to receive RDN.^12,13^ At first observation, 12-month results seem to mirror those from 6 months, with significantly greater office BP reductions for RDN patients compared with sham control patients, but with similar 24-hour ambulatory BP treatment differences between groups.^10^ However, these observations deviate from 6-month results in the context of a significantly greater antihypertensive medication burden among sham control patients, reflecting changes in medication number, dose, and class after the unblinding period. Thus, at the 12-month follow-up, RDN patients achieved comparable BP reductions as the sham control group despite a significantly lower medication burden confirmed by adherence testing. By 24 months, the number of antihypertensive medications remained higher in the sham control group, although the medication burden was similar between groups. However, RDN patients had significantly greater office and 24-hour ambulatory BP reductions. The persistence of BP reduction is also evident in the hourly ambulatory BP measures with RDN patients demonstrating clinically meaningful and more consistent reductions compared with sham control patients throughout the 24-hour period. In addition to reductions in morning and nighttime BP that are associated with higher risk, the greater stability in BP control after RDN warrants further study to demonstrate improvements in clinical outcomes. Whether progressive declines in BP after RDN are attributed to resetting of neurohormonal and sympathetic activity or to vascular remodeling is uncertain, but they are at least consistent with preclinical studies demonstrating the absence of functional nerve reinnervation.^21–23^ Independent of medication or lifestyle changes, the continued reductions in BP with RDN over extended follow-up are also consistent with a recent pooled analysis of 2016 patients with >3-year follow-up demonstrating durable BP reductions without an increase in antihypertensive medication number.^24^

As the most direct comparison of late-term outcomes, the primary analysis was limited to those patients randomized to treatment assignment and without crossover through 2 years. However, approximately one-half of patients assigned to the sham control group crossed over to treatment with RDN after 6 months. Follow-up in crossover patients through 24 months is still ongoing. Importantly, patients were permitted to cross over without requalifying BP criteria, and therefore had more variable BP at later time due to medication changes. To account for missing BP values from sham control patients who crossed over to receive RDN, we conducted several sensitivity analyses common to RDN studies, including imputation using the last observation carried forward.^12,13^ Such challenges are not unique to RDN studies.^25^ Overall, the clinically meaningful and significantly greater systolic BP reductions among RDN patients compared with control patients through 24 months, with or without imputation, support the efficacy of RDN through long-term follow-up.

The present findings contribute to the long-term safety associated with radiofrequency RDN using the Symplicity SPYRAL device. Consistent with previous studies of radiofrequency RDN with long-term follow-up, adverse clinical events were uncommon and did not differ between groups.^12,13,18,19^ Similarly, observed changes in eGFR were within an expected range and remained similar between treatment groups. Among patients receiving RDN, no occurrence of renal artery stenosis was reported by either protocol-mandated imaging at 6 months or during clinical follow-up.

### Limitations

Important limitations to this prespecified long-term analysis exist. Because patients and treating staff were no longer blinded after the primary end point ascertainment, and antihypertensive medication adjustments were permitted per protocol, unmeasured confounding factors are possible. Moreover, patients were aware of the trial end points and could influence results via self-modification of antihypertensive medication intake.^10,11^ This, however, is at least in part mitigated by medication adherence testing. Nevertheless, the performance of testing at discrete time points does not necessarily imply complete adherence or nonadherence over an extended period. This trial was not statistically powered to assess the efficacy of RDN versus a sham control procedure among different races or in patients with other comorbidities. However, there is no physiological evidence to suggest differential effects of RDN in other racial backgrounds. Additionally, the number of sham control patients with available long-term follow-up through 24 months was reduced due to patient crossover. To address this limitation, multiple sensitivity analyses were performed comparing BP reductions between the RDN group and the sham control group, showing similar results to the primary analysis. All sham control patients were allowed to cross over and did not have to requalify based on the original trial criteria to cross over. Indeed, one-half of crossover patients were no longer hypertensive at the time of their RDN procedure, possibly biasing last observations carried forward imputation in favor of sham. Multiple imputation analysis assumes data are missing at random, which is not necessarily the case. Ambulatory and office BP measures were not available for some sham control patients due to the initial design of the Pilot trial that only required phone visits after a 12-month follow-up.^26^ Finally, the results observed with this therapy and in this specific population may not be generalizable to alternative interventional therapies for hypertension and more varied clinical populations.

### Conclusions

In this prespecified analysis of long-term results of the SPYRAL HTN-ON MED trial comparing BP and medication changes between RDN and sham control patients, RDN provided clinically meaningful and significantly greater office and 24-hour ambulatory systolic BP reductions compared with the sham control procedure. Adverse clinical events were rare, and there were no confirmed instances of renal artery stenosis. These results support the growing body of evidence of the durable efficacy and safety of radiofrequency RDN as an additional therapeutic pillar to lifestyle modifications and antihypertensive medications in the treatment of hypertension.

## Article Information

### Acknowledgments

Benjamin Woods, PhD, of Medtronic, provided editorial support under the direction of the first author, including the creation of tables, figures, and editing of text. In collaboration with the funder, the executive committee designed the protocol and identified suitable clinical sites to conduct the study. Medtronic was responsible for data collection, monitoring, and analysis. The article was written by the lead author with contributions from coauthors. The funder assisted the authors in figure and table generation and curation, copy editing, and formatting. All authors had full access to the data and were responsible for the decision to submit for publication.

### Sources of Funding

This study was funded by Medtronic.

### Disclosures

Dr Kandzari discloses receiving institutional research/grant support from Biotronik, Boston Scientific, OrbusNeich, Teleflex, Medtronic, and Ablative Solutions; he also receives personal consulting honoraria from Ablative Solutions, Medtronic, and HyperQure. Dr Mahfoud has been supported by Deutsche Forschungsgemeinschaft (SFB TRR219, Project ID 322900939) and Deutsche Herzstiftung. Saarland University has received scientific support from Ablative Solutions, Medtronic, and ReCor Medical. Until May 2024, Dr Mahfoud has received speaker honoraria/consulting fees from Ablative Solutions, AstraZeneca, Inari, Medtronic, Merck, Novartis, Philips, and ReCor Medical. Dr Townsend is a consultant for Medtronic, Axio, Regeneron, Bard, OBIO, and AstraZeneca. Royalties from UpToDate. Dr Kario receives personal fees from Medtronic, receives grants from A&D Company, JIMRO, Omron Healthcare, CureApp, Terumo, and Fukuda Denshi, receives honoraria from Otsuka Pharmaceuticals and Omron Healthcare, and participates on the advisory board of Fukuda Denshi outside the submitted work. Dr Weber has received consulting fees from Medtronic, ReCor Medical, Ablative Solutions, Johnson & Johnson, and Urovant. Dr Schmieder has received speaker and consulting honoraria from Medtronic, Recor, and Ablative Solutions. Research grants have been given to his institution from Medtronic, Recor, and Ablative Solutions. Dr Tsioufis reports institutional research/grant support from Medtronic and ReCor Medical, and personal consulting honoraria from AstraZeneca, Bayer, Boehringer Ingelheim, Medtronic, ReCor Medical, Servier, Win Medica, and ELPEN. Dr Pocock reports personal fees from Medtronic, Edwards Lifesciences, and Boston Scientific. Drs Liu and Brar are employees of Medtronic. V. DeBruin is an employee of Medtronic. Dr Böhm is supported by the Deutsche Forschungsgemeinschaft (SFB TTR219), receives personal fees from Abbott, Amgen, AstraZeneca, Bayer, Boehringer Ingelheim, Cytokinetics, Medtronic, Novartis, ReCor Medical, Servier, and Vifor.

### Supplemental Material

Tables S1–S5

Figures S1–S5

## Supplementary Material
