# Supplementary material for: Long-Term Safety and Efficacy of Renal Denervation: 24-Month Results From the SPYRAL HTN-ON MED Trial
Source: Circ Cardiovasc Interv. 2025 May 20;18(7):e015194. doi: 10.1161/CIRCINTERVENTIONS.125.015194 (PMC12244969; doi:10.1161/CIRCINTERVENTIONS.125.015194)
Supplement: Supplementary file 1 [file hcv-18-e015194-s001.pdf]

SUPPLEMENTAL MATERIAL

Supplemental Figures

Supplemental Figure S1. 24-h ambulatory and office diastolic BP changes through 24 months

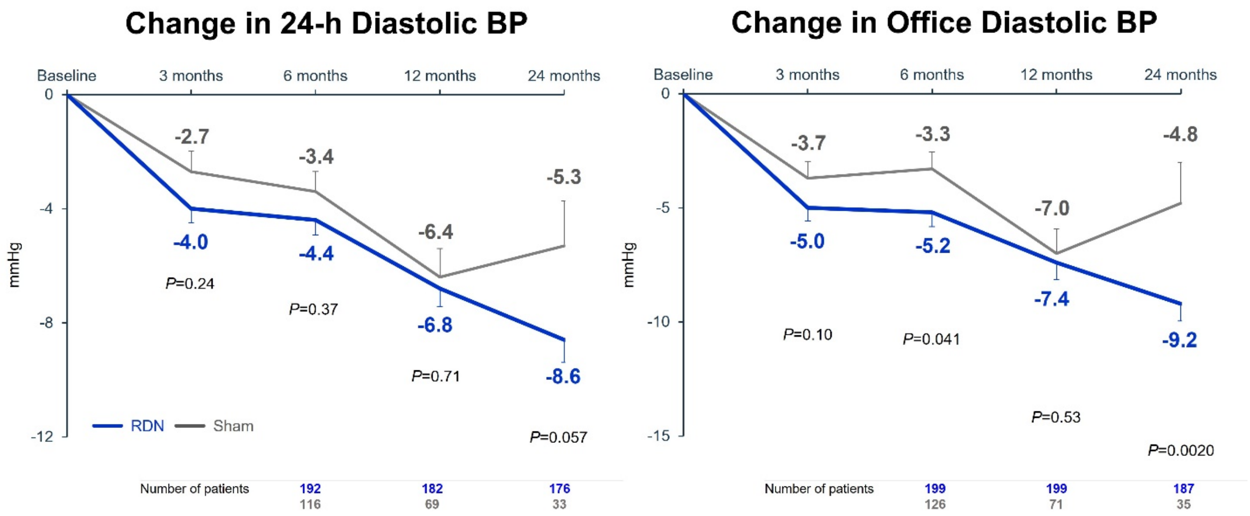

Treatment differences and p-values between RDN (blue) and sham groups (gray) are ANCOVA-adjusted for baseline BP.

Supplemental Figure S2. Hourly ambulatory systolic and diastolic BP through 24 months

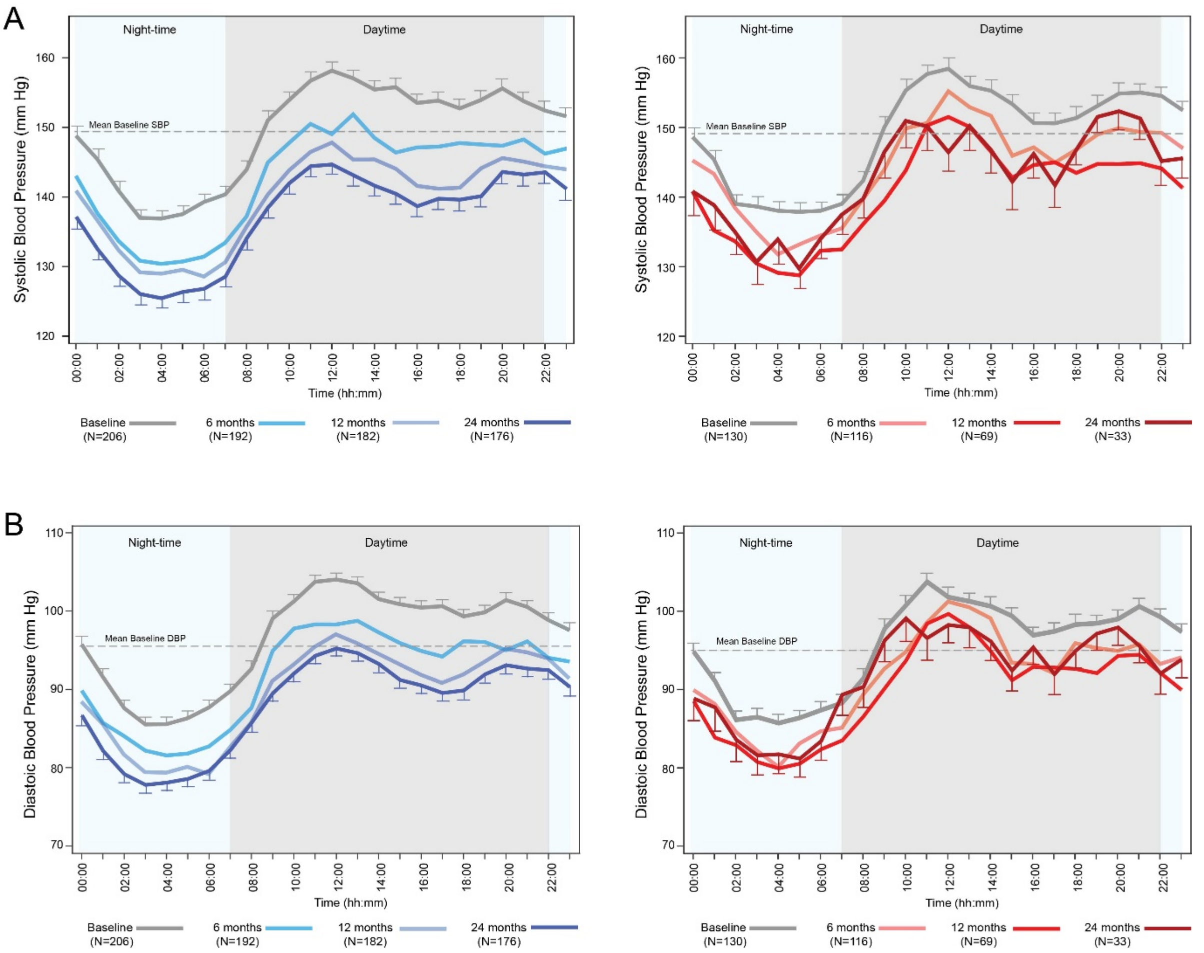

Hourly ambulatory (A) systolic and (B) diastolic BPs at baseline, 6 months, 12 months, and 24 months in the RDN (blue shades) and sham control groups (red shades).

Supplemental Figure S3. Difference in hourly ambulatory systolic BP from baseline to 24 months between RDN and sham control groups

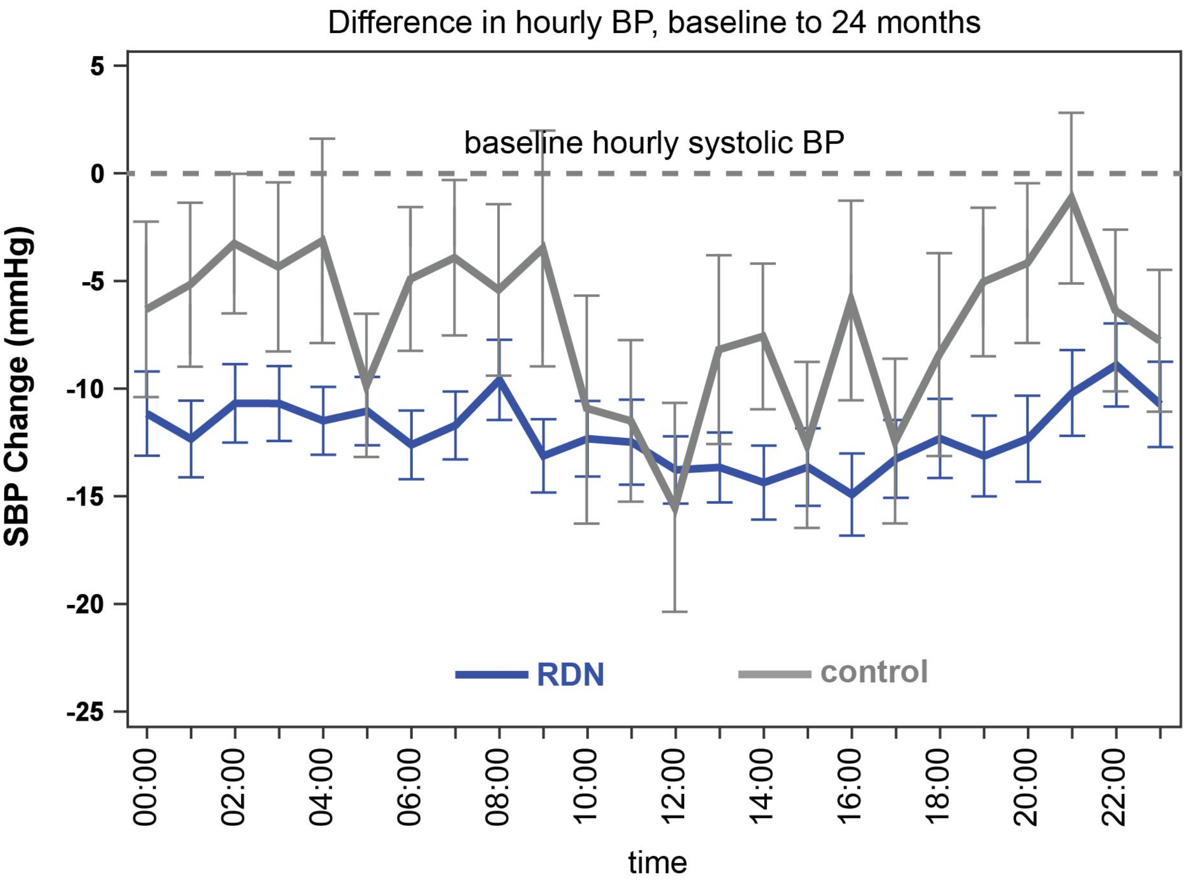

The difference in hourly BP from 24-month follow-up from baseline BP are plotted for RDN (blue) and sham control groups (red). Error bars are standard error.

Supplemental Figure 4. Twenty-four hour systolic BP and office systolic BP Forest plots comparing RDN vs. sham control at 24 months of specified subgroups

Subgroup		RDN N	Sham N	24-h ambulatory systolic BP adjusted treatment difference mmHg (95% CI)	interaction p-value
**Sex**	Male	146	29		**0.40**
	Female	30	4		
**Age**	< 65	147	28		**0.77**
	≥ 65	29	5		
**BMI**	Tertile 1 (<28.9)	69	11		**0.23**
	Tertile 2 (28.9 to 33.1)	56	11		
	Tertile 3 (≥33.1)	51	11		
**Type 2 Diabetes**	Yes	19	4		**0.95**
	No	157	29		
**Geography**	U.S.	73	11		**0.24**
	Outside U.S.	103	22		
**Race (within U.S.)**	Black American	29	6		**0.16**
	Non-Black American	44	5		
**Baseline 24-Hour SBP by Tertiles**	1: < 145	58	7		**0.63**
	2: 145 – 152	55	15		
	3: ≥ 152	63	11		

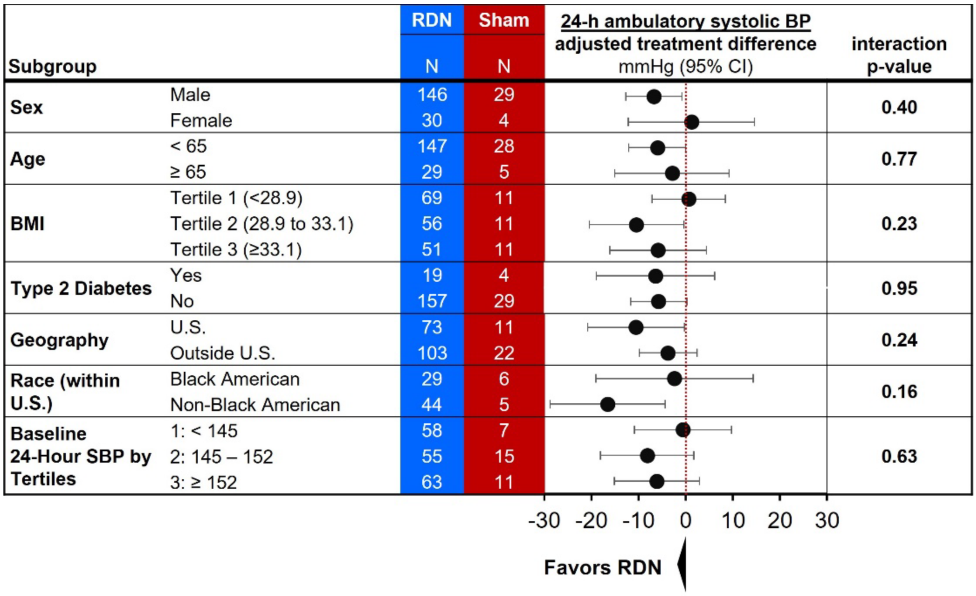

Subgroup		RDN N	Sham N	Office systolic BP adjusted treatment difference mmHg (95% CI)	interaction p-value
**Sex**	Male	154	30		**0.58**
	Female	33	5		
**Age**	< 65	157	30		**0.33**
	≥ 65	30	5		
**BMI**	Tertile 1 (<28.9)	72	12		**0.010**
	Tertile 2 (28.9 to 33.1)	58	11		
	Tertile 3 (≥33.1)	57	12		
**Type 2 Diabetes**	Yes	20	5		**0.85**
	No	167	30		
**Geography**	U.S.	81	13		**0.22**
	Outside U.S.	106	22		
**Race (within U.S.)**	Black American	31	7		**0.37**
	Non-Black American	50	6		
**Baseline Office SBP by Tertiles**	1: < 159	65	15		**0.22**
	2: 159 – 166	60	7		
	3: ≥ 166	62	13		

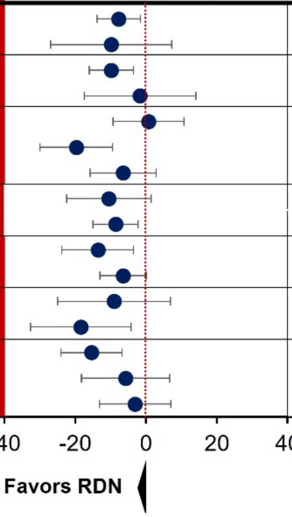

BP differences were ANCOVA adjusted for baseline BP and treatment type. BMI = body mass index.

Supplemental Figure S5. The change systolic BP measures at 24 months from baseline between RDN and sham control patients imputed by Last Observation Carried Forward for missing BP values

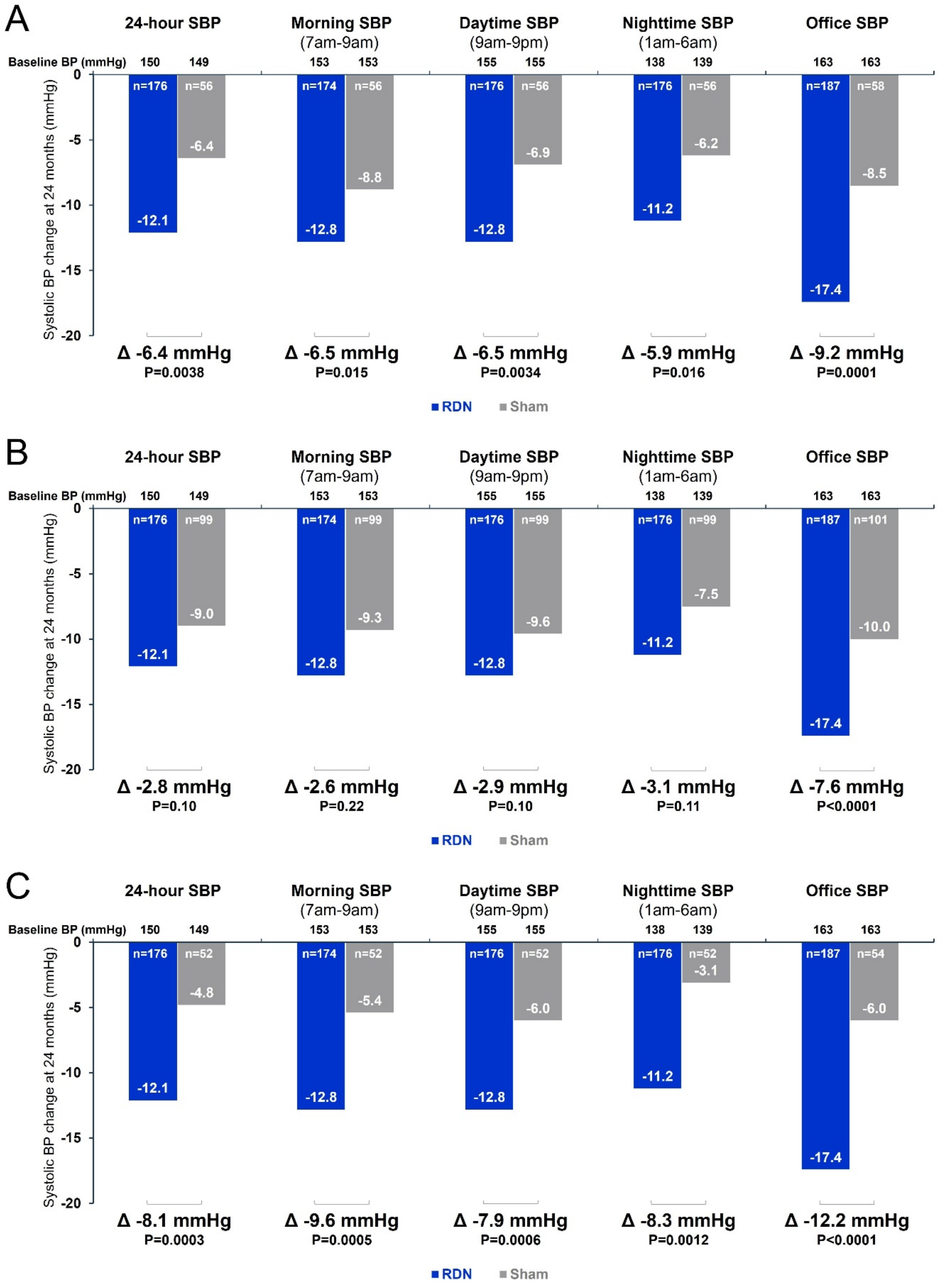

Imputation by Last Observation Carried Forward (LOCF) of sham control patients with missing BP values at 24-month follow-up: A) sham control patients with missing 24-month BP values due their final follow-up being conducted as phone visits (n=23); B) all sham control patients who crossed over to receive RDN before their 24-month follow-up (n=66); C) only sham control patients who crossed over to receive RDN before their 24-month follow-up who remained hypertensive (office systolic BP ≥150 mmHg and 24-h systolic BP ≥140 mmHg) at the time of crossover (n=19). SBP = systolic

Supplemental Tables

Supplemental Table S1. Secondary safety and efficacy endpoints

Secondary safety endpoints

Acute/procedural safety at 1-month post-procedure:

- Significant embolic event resulting in end-organ damage
- Renal artery perforation/dissection requiring intervention
- Vascular complications
- End stage renal disease
- ≥40% decline in estimated glomerular filtration rate
- Increase in serum creatinine >50% from baseline
- New Myocardial Infarction
- New Stroke
- Major bleeding according to TIMI definition
  - Intracranial hemorrhage
  - ≥5g/dl decrease in hemoglobin concentration
  - ≥15% absolute decrease in hematocrit
  - death due to bleeding within 7 days of the procedure
- New renal artery stenosis >70% confirmed by angiography and as assessed by angiographic core laboratory
- Hospitalization for hypertensive crisis not related to confirmed non-adherence with medications/protocol

Long-term safety through 24 months post-procedure:

- All-cause mortality
- Myocardial infarction
- Major bleeding (TIMI)
- Significant embolic event resulting in end-organ damage,
- Renal artery re-intervention
- Vascular complications requiring surgical repair, intervention, thrombin injection, or blood transfusion
- Hypertensive crisis,
- Stroke
- Renal artery stenosis >70%

Secondary efficacy endpoints (3, 6, 12, 24, and 36 months)

- Change in 24-h ambulatory systolic blood pressure
- Change in office systolic blood pressure
- Change in 24-h ambulatory diastolic blood pressure
- Change in office diastolic blood pressure
- Incidence of achieving target office systolic blood pressure (<140 mm Hg)

Supplemental Table S2. Proportion of patients with indicated reductions in systolic BP at 2 years

Reductions in office systolic BP	renal denervation	sham control	P-value
≥5 mmHg	80% (150/187)	63% (22/35)	0.029
≥10 mmHg	73% (136/187)	51% (18/35)	0.016
≥15 mmHg	56% (104/187)	34% (12/35)	0.026
≥20 mmHg	44% (82/187)	26% (9/35)	0.060
**Reductions in 24-h ambulatory systolic BP**	**renal denervation**	**sham control**	**P-value**
≥5 mmHg	74% (130/176)	55% (18/33)	0.025
≥10 mmHg	59% (103/176)	39% (13/33)	0.042
≥15 mmHg	44% (77/176)	36% (12/33)	0.43
≥20 mmHg	32% (57/176)	18% (6/33)	0.10

Supplemental Table S3. The change systolic BP measures at 12 and 24 months from baseline in pooled RDN and crossover patients versus sham control non-crossover patients

	Time point [a]	RDN and crossovers [b,c]	Non-crossovers	p-value [d]	p-value [e]
24-h Ambulatory	Baseline	149.3 ± 6.9 (N=272)	150.3 ± 7.4 (N=64)	0.27	0.27
	Change at 6 months	-7.9 ± 11.6 (N=251)	-3.6 ± 11.5 (N=55)	0.0075	0.0063
	Change at 12 months	-10.7 ± 12.7 (N=243)	-7.8 ± 12.1 (N=57)	0.081	0.044
	Change at 24 months	-12.1 ± 15.3 (N=176)	-7.0 ± 13.1 (N=33)	0.039	0.043
Office	Baseline	163.0 ± 7.8 (N=272)	163.0 ± 7.4 (N=65)	>0.99	>0.99
	Change at 6 months	-11.3 ± 15.0 (N=262)	-5.2 ± 13.0 (N=62)	0.0027	0.0023
	Change at 12 months	-15.5 ± 16.4 (N=263)	-10.9 ± 15.0 (N=59)	0.038	0.023
	Change at 24 months	-17.4 ± 16.1 (N=187)	-9.0 ± 19.4 (N=35)	0.0034	0.0034

Data are represented as mean ± SD mmHg (N). Baseline values are at the time of randomization. Crossover patients did not have to re-qualify to undergo the RDN procedure. [a] Blood pressure change at the indicated time point is post-procedure for crossover patients. [b] The mean time for crossover post-randomization was 241 ± 86 days (median: 201 days). [c] Crossover data is reported through 12 months post-procedure. [d] P-values are ANCOVA-adjusted for baseline blood pressure. [e] P-values are ANCOVA-adjusted for baseline blood pressure and the change in medication burden.

Supplemental Table S4. The number of antihypertensive medications and medication burden in crossover and non-crossover patients from the sham control group from baseline through final follow-up

Number of AH medications	Crossover (n=66)	Non-crossover (n=65)	Difference	P-value
Baseline	1.8 ± 0.7	2.1 ± 0.9	-0.4	0.010
6 months	1.9 ± 0.8	2.3 ± 0.9	0.04	0.63
Change from baseline to 6 months	0.2 ± 0.5	0.1 ± 0.3	0.04	0.63
Final follow-up*	2.3 ± 0.9	2.7 ±1.1	-0.2	0.27
Change from 6 months to final follow-up	0.4 ± 0.7	0.5 ± 1.1	-0.3	0.097
**AH medication burden**	**Crossover**	**Non-crossover**	**Difference**	**P-value**
Baseline	2.5 ± 3.0	3.0 ±3.5	-0.5	0.40
6 months	2.9 ± 2.7	3.6 ± 3.4	-0.3	0.40
Change from baseline to 6 months	0.4 ± 2.9	0.6 ± 1.9	-0.3	0.40
Final follow-up*	4.0 ± 3.8	6.1 ± 5.6	-1.6	0.031
Change from 6 months to final follow-up	1.0 ±3.0	3.1 ± 5.1	-2.2	0.0039

*Final follow-up for crossover patients is the final BP measure available after their crossover procedure (12 months post-crossover), whereas the final follow-up for non-crossover patients is their last available BP measure since enrollment and randomization to the control group (24 months post-randomization).

Supplemental Table S5. Safety events at 24 months

% (n)	Denervation (N=206 Subjects)	Sham Group (N=131 Subjects)
Composite Safety Endpoint[1]	2.5% (5)	2.6% (2)
Death	0.5% (1)	1.8% (1)
New MI	0.0% (0)	0.0% (0)
Major Bleeding (TIMI)	0.0% (0)	0.0% (0)
Significant embolic event resulting in end-organ damage	0.5% (1)	0.0% (0)
Renal artery re-intervention	0.0% (0)	0.0% (0)
Vascular complications requiring surgical repair, interventional procedure, thrombin injection, or blood transfusion	1.0% (2)	0.8% (1)
Hospitalization for hypertensive crisis/emergency	0.5% (1)	0.0% (0)
New Stroke	0.5% (1)	0.8% (1)
New Renal Artery Stenosis > 70%	0.0% (0)	0.0% (0)

Estimate adverse event rates from Kaplan-Meier curves through 24 months. [1] Composite safety endpoint is all cause mortality, end-stage renal disease, significant embolic event resulting in end-organ damage, renal artery perforation requiring intervention, renal artery dissection requiring intervention, vascular complications, hospitalization due to a hypertensive crisis not related to confirmed non-adherence with medications and/or the protocol, and new renal artery stenosis >70% confirmed by angiography determined by the angiographic core laboratory.
